# Fantastic AAV Gene Therapy Vectors and How to Find Them—Random Diversification, Rational Design and Machine Learning

**DOI:** 10.3390/pathogens11070756

**Published:** 2022-07-03

**Authors:** Jonas Becker, Julia Fakhiri, Dirk Grimm

**Affiliations:** 1 Department of Infectious Diseases/Virology, Medical Faculty, University of Heidelberg, Center for Integrative Infectious Diseases Research (CIID), BioQuant, 69120 Heidelberg, Germany; jonas.becker@bioquant.uni-heidelberg.de; 2 Faculty of Biosciences, University of Heidelberg, 69120 Heidelberg, Germany; 3 Roche Pharma Research and Early Development, Therapeutic Modalities, Roche Innovation Center Munich, Roche Diagnostics GmbH, Nonnenwald 2, 82377 Penzberg, Germany; 4 German Center for Infection Research (DZIF), Partner Site Heidelberg, 69120 Heidelberg, Germany; 5 German Center for Cardiovascular Research (DZHK), Partner Site Heidelberg, 69120 Heidelberg, Germany

**Keywords:** adeno-associated virus, AAV, gene therapy, molecular evolution, capsid engineering

## Abstract

Parvoviruses are a diverse family of small, non-enveloped DNA viruses that infect a wide variety of species, tissues and cell types. For over half a century, their intriguing biology and pathophysiology has fueled intensive research aimed at dissecting the underlying viral and cellular mechanisms. Concurrently, their broad host specificity (tropism) has motivated efforts to develop parvoviruses as gene delivery vectors for human cancer or gene therapy applications. While the sum of preclinical and clinical data consistently demonstrates the great potential of these vectors, these findings also illustrate the importance of enhancing and restricting in vivo transgene expression in desired cell types. To this end, major progress has been made especially with vectors based on Adeno-associated virus (AAV), whose capsid is highly amenable to bioengineering, repurposing and expansion of its natural tropism. Here, we provide an overview of the state-of-the-art approaches to create new AAV variants with higher specificity and efficiency of gene transfer in on-target cells. We first review traditional and novel directed evolution approaches, including high-throughput screening of AAV capsid libraries. Next, we discuss programmable receptor-mediated targeting with a focus on two recent technologies that utilize high-affinity binders. Finally, we highlight one of the latest stratagems for rational AAV vector characterization and optimization, namely, machine learning, which promises to facilitate and accelerate the identification of next-generation, safe and precise gene delivery vehicles.

## 1. Introduction

Parvoviruses are small, non-enveloped viruses that belong to the family *Parvoviridae*. They infect both vertebrates and invertebrates and are composed of an icosahedral capsid carrying a single-stranded DNA genome of 4.7–6 kb in size. More than three decades ago, researchers recognized the potential of parvoviruses in medical applications that either (i) depend on properties of the wild-type (WT) virus itself, such as the ability of autonomous protoparvoviruses to replicate in cancer cells [1], or that (ii) utilize recombinant viruses or so-called vectors, which lack part of or all viral elements (the latter are called gutless vectors) and instead harbor a transgene of interest [2,3]. This review will focus on the latter type and more specifically highlight the Adeno-associated virus (AAV) that serves as a template for intensive preclinical research and has been employed as delivery platform in over 250 clinical trials [4]. Gene therapy with AAV vectors has consistently demonstrated their immense potential and general safety, but has also raised critical concerns. In particular, many of the first-generation vectors showed insufficient on-target potency and/or specificity or high levels of adverse off-targeting, which necessitated the application of high doses. These high vector doses have recently been associated with serious adverse events in patients, including multiple fatalities in children in a trial employing AAV8 vectors for the treatment of myotubular myopathy [5]. The lack of specificity and the ensuing consequences triggered efforts to screen for and develop new vectors with improved targeting properties and thus enhanced safety.

To this end, there are multiple options that act primarily on the level of the vector cargo (i.e., the recombinant viral genome) [6,7,8] and that will briefly be mentioned here, albeit they are not in the focus of this article. One powerful and widely employed technology is the use of tissue- or cell-type-specific promoters to drive and restrict AAV vector transgene expression in a desired target. While these promoters tend to be leaky, they can minimize unwanted vector expression in off-targets and thereby increase in vivo vector safety, especially when used in combination with capsids with a broad cell specificity. Clinically relevant examples for the use of such tissue-specific promoters include for example, HLP-promoter-driven expression of human blood coagulation factor VIII in the liver for AAV5-based treatment of hemophilia A (NCT03370913) [9], or the use of the muscle-specific CK8 promoter to drive mini-dystrophin expression from AAV9 for treatment of Duchenne muscular dystrophy (NCT03368742) [10]. Alternatively or in combination with tissue-specific promoters, another option to restrict AAV transgene expression is the addition of mi(cro)RNA binding sites in the 3′ untranslated region (UTR) of the expression cassette [11]. A prominent example that has been studied repeatedly and successfully before is miR-122, which is highly and specifically expressed in hepatocytes in the liver [12,13]. Accordingly, if an AAV vector genome is tagged with one or more binding sites for this miRNA, transgene expression will be diminished in the liver. In turn, this will increase specificity in all cases where the liver is considered as an unwanted target and where the AAV capsid and/or promoter are insufficiently specific to exclude expression in this off-target.

While the use of regulatory elements such as promoters or miRNA binding sites is a powerful strategy to improve vector specificity on the transcriptional or post-transcriptional level, respectively, the control of vector biodistribution on the transductional level requires the use of tissue-specific capsids. This specificity may be achieved by enforcing capsid binding to cell-type-specific surface receptors, or by modifying intracellular steps from cytoplasmic trafficking up to capsid uncoating in a cell-type-specific manner [14]. In this review, we will focus on such modifications of the viral capsid as a versatile means to generate AAV gene therapy vectors with increased on-target specificity and efficiency.

Notably, this strategy critically depends on detailed knowledge of the exact target tissues and cell types involved in a given disease. In this respect, next-generation technologies such as single-cell RNA sequencing have been very beneficial and increased the pace of disease-to-target/-vector discovery enormously [15,16]. This has not only enabled the discovery of new cell types but has also aided in deciphering their contribution to human disease progression. Accordingly, it has become increasingly clear that traditional approaches of vector screenings have their limitations, as they were often performed on the level of a whole organ and/or did not involve a parallel assessment of off-targeting. Examples for the involvement of specific cell types in disease pathogenesis are manifold and comprise e.g., hepatic stellate cells that are critical targets in liver fibrosis [17], polygonal cells of the retinal pigment epithelium (RPE) in macular degeneration [18], and dopaminergic neurons in Parkinson’s disease [19]. This also applies to monogenic diseases, for which AAV vectors serve as a platform of choice. One example is Duchenne muscular dystrophy (DMD), in which the skeletal and cardiac muscle cells are affected and less able to produce dystrophin due to a mutation in the DMD gene. In particular, the progenitors of skeletal muscle cells, the satellite cells, have been proposed as target cells for a promising and lasting DMD gene therapy approach [20]. Another example is cystic fibrosis, an inherited multiorgan disease that causes severe damage to the lungs. It has been recently shown that ionocytes, which are present at a low percentage in the lungs, express the highest level of the cystic fibrosis transmembrane conductance regulator (CFTR), which might render them important targets for therapeutic intervention [21].

Consequently, several research groups have already aimed to enrich cell-type-specific AAV capsids in high-throughput screens by e.g., utilizing cell-type-specific promoters [22,23] or Cre-transgenic mice that express the Cre recombinase in different cell types (e.g., the M-CREATE system) [24], as further discussed below. However, the isolation of specific cells out of complex cellular structures can be technically challenging as it typically depends on the availability of cell-surface markers for cell sorting. Alternatively, the identification of specific cell types in a complex mixture can be assisted by single-cell RNA sequencing [25]. Yet, this technology has not been used for directed AAV evolution thus far but instead only for stratification of pre-selected capsid variants [26,27,28]. Moreover, regardless of the technology used for cell and AAV isolation, the translatability of the isolated candidates to higher species is not always given. Another way to target specific cells is to make use of our knowledge about receptors and pathways to engineer ligands, antibodies, or small peptides that can be genetically or chemically coupled to the viral capsid [29,30,31,32]. While these rational approaches allow one to circumvent complicated and time-consuming high-throughput screens, their success is limited by the information on targeting molecules and target cells. Finally, data available from all these experimental strategies and basic biology research can be now used as a valuable resource for bottom-up approaches that might, in the future, allow for the in silico or in vitro creation of AAV capsids from scratch.

In the following, we will first provide an overview of the state-of-the-art unbiased and directed capsid-engineering approaches that have historically and recently been pursued to generate novel, more specific AAV vectors. Next, we highlight two of the latest avenues in rational bioengineering utilizing nanobody (Nb)- or Designed Ankyrin Repeat Protein (DARPin)-mediated AAV retargeting and critically assess their potential for future clinical applications. We finally discuss the merits of machine learning as a newly emerging technology that might revolutionize our way of vector design by enabling previously unprecedented levels of capsid precision, viability and efficacy.

## 2. Capsid Engineering to Replace or Expand Natural AAV Serotypes

**Figure 1:**
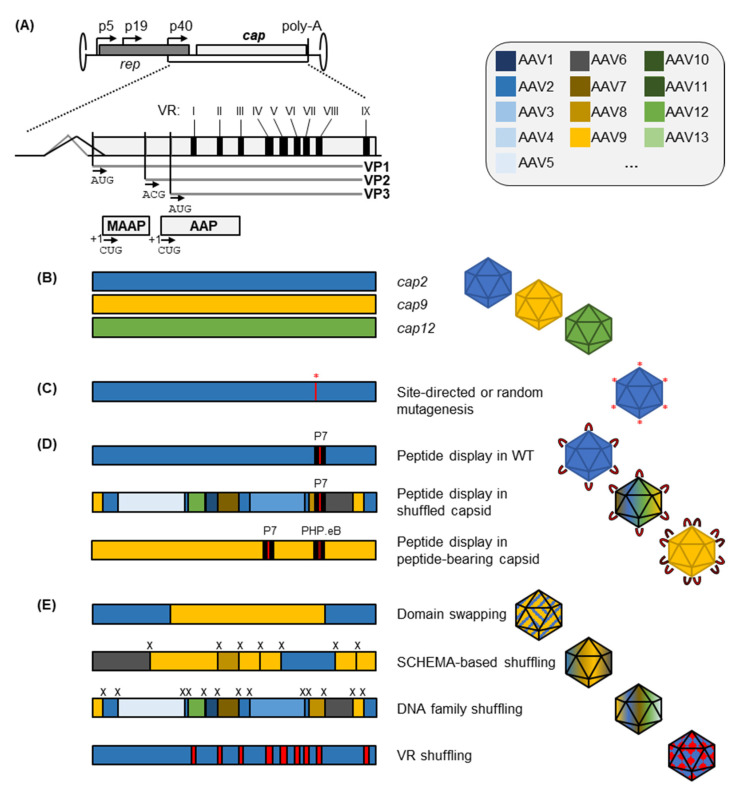
Structure of the AAV *cap* gene and technologies for its diversification. (**A**) Schematic of the AAV *cap* gene including variable regions (VRs I-IX according to Govindasamy et al. [36]) and transcriptional start sites for VP1, VP2 and VP3, as well as MAAP [37] and AAP [38]. p5, p19 and p40 are the endogenous AAV promoters. poly-A, polyadenylation signal. (**B**) Tropisms of AAV vectors can be defined by choosing one of 13 primate AAV serotypes (AAV1-13) or a plethora of other naturally occurring isolates from various species. (**C**) Wild-type tropisms can be modified by mutagenesis of one or several capsid residues (e.g., Kern et al. [39]). (**D**) Insertion of pre-defined or randomized peptide sequences (e.g., a randomized 7 mer peptide “P7”; red indicates the peptide sequence and black the flanking residues, such as glycine or alanine that can be used as linkers) can be performed within WT *cap* backbones (e.g., Müller et al. [40]), in synthetic capsids such as shuffled variants (e.g., Tan et al. [41]), or in backbones already carrying an independent peptide insertion in another position (e.g., Goertsen et al. [42]). The colors of the individual capsid fragments denote the serotype origin according to the legends in the upper right corner of this figure. (**E**) Recombination of larger *cap* stretches from several parental capsids can be performed via domain swapping (e.g., Shen et al. [43]), SCHEMA-based shuffling through pre-defined optimal crossover points (marked with “x”) (e.g., Ojala et al. [44]), DNA family shuffling based on partial sequence homology (e.g., Grimm et al. [45]), or virtual VR shuffling (e.g., Marsic et al. [46]).

While the commercially available AAV products, namely, Luxturna^®^ and Zolgensma^®^, are based on naturally occurring AAV variants, clinical studies have highlighted the limitations of, and concerns about, wild-type virus-derived vector variants [33,34]. These handicaps include the high levels of pre-existing neutralizing anti-AAV antibodies (NAbs) in the human population [11,35], which restrict the inclusion of patients or limit gene transfer. Other notable hurdles are the inept transduction profiles with unspecific tropisms and pronounced off-target transduction that consequently reduce the therapeutic benefit and require the application of high doses. To circumvent these pitfalls, capsid modification strategies can be employed that aim to create synthetic derivatives of naturally occurring AAVs with enhanced specificity, immune escape, and safety profiles. Examples of these modifications are schematically represented in Figure 1 and will be discussed in the following sections.

Most of the isolated AAV serotypes and other natural variants reported to date exhibit a broad, non-specific transduction profile upon systemic administration with a bias towards the liver and spleen [47,48]. It is, however, important to mention that the route of administration plays a critical role in defining the tropism of a vector as well, next to the nature of the capsid. For example, the Anc80L65 capsid predominantly transduces the liver when applied systematically [49] but shows distinct transduction profiles in the inner ear upon local administration [50]. The vector of choice should therefore be selected and optimized depending on the tissue or cellular target of interest and the application route, next to other considerations such as a patient’s anti-AAV immune status. For systemic applications, efficient targeting often requires an engineering effort to allow sufficient vector homing to the intended target cells.

Various strategies have been employed to alter the properties of AAV capsids including modification of their inherent tropisms. These strategies can be roughly classified into (i) rational design of modified capsid structures and (ii) randomized modifications that are combined with directed evolution in order to enrich capsid variants with desired features [51,52]. Both philosophies have inherent flaws: rational design is always limited by the understanding of the viral capsid and its host interactions, as well as the knowledge about potentially useful receptor interactions for implementation into capsid retargeting. Directed evolution, on the other hand, is limited by the quality of the input library of capsid variants from which to select, and the interplay of positive and negative selection pressures, which have to be carefully chosen to force the enrichment of desired capsid features. Moreover, irrespective of methodology, there is always the possibility that alterations in AAV capsid sequence and structure will not only change the transduction properties of the engineered capsid but also impact its ability to assemble and package viral genomes. Since these parameters critically determine the ultimate applicability of synthetic AAV capsids in human patients, they have to be monitored carefully during capsid engineering, and libraries should be optimized for vitality (so-called “smart libraries”) rather than for mere complexity. The latter is often represented in the literature by the numbers of bacterial colonies that were counted after transformation of a plasmid library pool (e.g., 1 × 10^8^), yet this value does not necessarily reflect the infectivity of a library and the proportion of functional capsids. For a more in-depth discussion of this complex topic, we refer the reader to more dedicated former review articles [52,53].

While neither approach at AAV capsid engineering is perfect, each has delivered impressive results, and the strategies employed in the two avenues are often mutually beneficial. As the number of these methodologies is constantly increasing, we can merely highlight representative examples of capsid optimization and diversification strategies in the following, rather than comprehensively review the entire field of AAV capsid engineering. Hence, we apologize to our numerous colleagues whose pivotal research on other aspects of AAV vector evolution, such as advances in library selection schemes and improvements in vector tracking, we had to exclude from this review due to limited space. Instead, we refer the readers to recent review articles published elsewhere that comprehensively cover these other topics including AAV vector applications [34,54,55], AAV library selection [52,56], and AAV vector design in general [57,58].

### 2.1. Peptide Insertion or Replacement for AAV Capsid Retargeting

Modifications of the AAV capsid were initially applied to AAV2 as the “AAV workhorse”, which was the first AAV cloned as an infectious virus [59] and is therefore most thoroughly characterized. Re-targeting of the AAV2 capsid was attempted early on by insertional or site-directed mutagenesis [60,61,62,63]. This demonstrated that the AAV capsid tolerates small insertions of peptide ligands at sterically convenient positions on the capsid surface. Pioneering work by Girod et al. identified these insertion sites by aligning the AAV2 capsid amino acid sequence to the ones from canine parvovirus, for which the X-ray crystal structure was already available [60]. Insertion of the 14-amino-acid L14 peptide identified two insertion sites, 447 and 587 (VP1 numbering), that would still allow proper capsid assembly plus L14-mediated integrin receptor binding. Solving AAV2′s crystal structure later confirmed these two positions to lie within the two highest protruding surface loops of the AAV capsid, located around the three-fold axis of symmetry [64]. An improved understanding of potential acceptor sites has then resulted from comparison of AAV2 to the structure of AAV4, which is one of the most diverse of all AAV serotypes [36]. This demonstrated the presence of nine surface-oriented protein loops on the AAV capsid, which were termed variable region (VR) I–IX due to their low level of conservation between the serotypes. AAV2′s ability to bind heparan sulfate proteoglycans (HSPG) had been located to lysine and arginine residues including R585 and R588 [39,65] within VR VIII. Mutating these sites by amino acid substitution or peptide insertion prevents this receptor interaction and thus enables vector detargeting from the liver and spleen, common off-targets in many gene therapy applications [66,67,68].

Currently, most insertions of peptide ligands are typically performed in variable regions IV (positions 453 in AAV2) and VIII (positions 587/588) [69]. A recent direct comparison between the two insertion sites for display of an insulin-mimetic peptide in AAV9 demonstrated a slightly better tolerance for insertion in VR IV, while better retargeting to the insulin receptor after intramuscular application was achieved through insertion in the VR VIII loop [31]. As in this example, inserted peptides can be selected as definite sequences with known interactions to improve the transduction of target cells expressing the respective receptors. However, the empirical selection of a peptide for insertion into an AAV capsid is always limited by the knowledge of a potentially useful ligand-receptor-interaction. In addition, even if a useful peptide ligand is known, its introduction into the AAV capsid is not guaranteed to yield functional capsids. To circumvent these limitations and to create novel tropisms with hitherto unknown receptor interactions, a more serendipitous strategy is required, such as directed evolution. A standard directed evolution approach for AAV capsid diversification is the insertion of random peptides into the previously identified capsid positions, followed by the use of in vitro or in vivo selection strategies to enrich beneficial variants. This approach has been adopted from phage display and was thus termed “AAV display” [40,70]. There are manifold successful examples demonstrating the power of randomized peptide display on AAV capsids. For instance, the AAV2 capsid has been evolved via peptide display for pulmonary targeting upon systemic injection in mice, which yielded an “ESGHGYF”-peptide bearing AAV2 capsid that was largely detargeted from liver and other off-target organs [71]. This was achieved by closely monitoring on- and off-target variant enrichment over multiple selection rounds via Next-Generation Sequencing (NGS). Another example of a modified AAV2 tropism is AAV2-7m8, a peptide display variant evolved to enable photoreceptor transduction in the retina after intravitreal administration [72]. Interestingly, work by Khabou et al. demonstrated that insertion of the 7m8 peptide similarly enhances the retinal transduction of the AAV9 capsid but did not exert such effects within an AAV5 or AAV8 background [73]. Thus, the capsid context of the selected peptide variant is of crucial importance and is not limited to the AAV2 capsid, as exemplified further in the next section.

#### 2.1.1. Peptide Display in Serotypes Other than AAV2

Although most insertional studies so far have been performed with the AAV2 serotype, other naturally occurring capsids may present more relevant baseline features, such as the inherent ability of AAV9 to cross the blood-brain barrier (BBB) [74] or the unique tropism of AAV6 for hard-to-transduce hematopoietic cells [75]. An elaborate insight into AAV peptide display in different capsid backgrounds was provided by Börner et al. [49] and Weinmann et al. [76]. In these studies, insertions of pre-defined peptides in 13 different capsids were guided by crystal structures of the respective VR VIII loops, and were tested for several different capsids in a multitude of cell types or organs, respectively. This approach revealed or confirmed several insights into AAV peptide display: (i) the functionality of an inserted peptide is strongly dependent on its capsid background, (ii) non-AAV2 capsids bear great potential for developing highly functional vectors upon peptide insertion, and (iii) peptide-flanking regions can strongly affect the overall phenotype. Several of these insights were mirrored between different peptide display studies. For instance, presentation of an RGD-bearing peptide in AAV9 has provided the resulting AAVMYO capsid with a unique muscle-targeting phenotype in mice [76]. A similar family of peptides was independently evolved in an AAV9 background by Tabebordbar et al., yielding a clade of AAV capsid variants termed MyoAAV [23]. Interestingly, concurrent evolution of the RGD-flanking amino acid residues yielded derivatives with preferred muscle transduction either in mice (MyoAAV 2A) or in cynomolgus macaques (MyoAAV 4A). The success of these peptide-bearing capsids is to some extent owed to improving the general features of their parental AAV9 capsid, which is known for its efficient and broad transduction profile and long persistence in the blood, allowing for *trans-*vascular and *trans-*endothelial vector transport [68,77]. This even grants it the ability to cross the blood-brain-barrier (BBB) at a low frequency, which is, however, overshadowed by its transduction of other organs [78,79]. Building upon this phenotype by adding variable peptide insertions and by in vivo screening for central nervous system (CNS)-transducing variants yielded capsids with the ability to efficiently induce homing across the BBB after systemic administration [22,80,81]. One prominent example of CNS-targeted transduction upon intravenous administration was reported by Deverman et al. [80], who performed AAV9-based peptide display screens using a Cre-based functional selection scheme that produced the AAV9-PHP.B capsid. In a follow-up study by Chan et al. [81], homing to CNS was improved by optimizing the amino acid residues flanking the PHP.B peptide, yielding the PHP.eB capsid with even lower off-target transduction. Intriguingly, the receptor interaction of PHP.B/PHP.eB, which was later identified as Ly6a [82,83], demonstrated efficient binding only for the Ly6a haplotype of the animal model (C57BL/6J mice) applied during capsid evolution, and thus did not translate to other mouse strains or non-human primates [84,85,86]. Yet another improvement in the PHP.B journey has recently been achieved via directed evolution, by inserting a randomized 7mer peptide into the VR IV loop of the AAV9-PHP.eB capsid [42]. Using the M-CREATE system, the library was screened in multiple Cre-transgenic C57BL/6J mice that express the Cre recombinase in different cell types. Monitoring the enrichment in on- and off-target tissues identified AAV.CAP-B10, a capsid providing strong transduction of CNS neurons and reduced off-targeting as compared to its parent AAV9-PHP.eB. Curiously, although the Ly6a haplotype is absent in non-human primates [87], the neurotropic phenotype of AAV.CAP-B10 translated extraordinarily well to marmosets and thus presents a pivotal progression for CNS gene therapy using intravenously injected vectors. The (M-)CREATE system and similar functional selection schemes focus on enriching variants based on nuclear transduction instead of mere accumulation of their encaspidated DNA within a target tissue or cell. Functional transduction can be assayed, for instance, by Cre-based recombination [24,44,80,88] or by driving *cap* gene expression from a ubiquitous or tissue-specific promoter to enable variant detection via the expressed RNA [22,23,89]. Therefore, capsid variants that achieve homing but fail at trafficking to the nucleus are not enriched during these functional screens. While these and other improvements have greatly improved the library selection and thus directed evolution of AAV capsids, we refer the reader to the original literature or more dedicated review articles owing to the aforementioned space reasons [52] (Szumska and Grimm, submitted).

#### 2.1.2. Non-Random Peptide Screens

As noted, most of the potent AAV peptide variants were originally selected from random peptide libraries displayed on the surface of AAV capsids, which were screened in a given organ or cell population. Some exceptions to the rule exist as comprehensively demonstrated by the above-mentioned studies of Börner et al. [49] and Weinmann et al. [76]. Another noteworthy example has recently been reported by Martino and colleagues, who aimed to transfer the BBB phenotype of AAV9-PHP.B to the AAV1 capsid by engrafting the PHP.B peptide [90]. Binding to Ly6a, however, was not achieved by merely inserting the peptide into VR VIII of AAV1, but required a concurrent transfer of the whole surrounding VR VIII loop from AAV9 onto AAV1. Still, this was not sufficient to enable crossing of the BBB, either due to lower Ly6a binding affinity, or due to the use of an inadequate capsid background incapable of transcytosis. As noted before, Khabou et al. followed a similar approach for AAV2-7m8 and were successful in transferring its retinal transduction phenotype onto AAV9 but not onto AAV5 or −8 [73]. Recently, the PHP.eB peptide [81] was rationally inserted into the shuffled AAV-DJ capsid [45] by Tan et al. in order to generate a cell-penetrating phenotype for the transduction of murine cochlear supporting cells [41]. From the ensuing AAV-ie capsid, a single-amino acid mutant (AAV-ie-K558R) has most recently been derived that exhibits robust transduction of outer hair cells in neonatal mice as well [91]. This vector enabled successful treatment of hearing loss in a mouse model and demonstrated that multiple iterations of engineering can be successfully combined within a single vector lineage through additive rational modifications. Finally, we point out work by Davidsson and co-workers, who performed AAV peptide display with the goal of achieving retrograde axonal transport upon injection into the striatum [92]. Instead of randomizing the peptide insert, peptide sequences were derived from viral and other protein sources capable of axonal transport. This allowed for the identification of lead candidates after only a single round of in vivo selection, yielding AAV2-peptide variants with enhanced CNS distribution through axonal transport.

While peptide display is a powerful tool for the creation of capsid variants with novel receptor interactions that might support retargeting to the intended target tissue, the engineered capsid will retain many of its parental features. Such features may be beneficial as demonstrated above for AAV9 but may also render the novel capsid vulnerable to the flaws inherent to its parental counterpart. For instance, peptide display does not mask most binding epitopes for NAbs. As these binding epitopes mostly map to the surface-exposed variable regions, a mutagenesis approach within these regions is a promising option for the creation of immune-evading capsids, as demonstrated by Tse et al. [93]. Specifically, NAb epitopes were mapped onto the AAV1 capsid, and a saturated mutagenesis of the respective regions in VR IV, V and VIII was combined with directed evolution of the resulting libraries. By employing iterative evolution and rational combination of enriched VR variants replacing the respective WT sequence at the interrogated VRs, an immune-evading variant of AAV1 called CAM130 was generated, which retains its parental biodistribution. Using cryo-electron microscopy-based identification of antibody-binding epitopes on the capsid surface, follow-up studies for AAV8 and AAV9 have been performed with similarly promising outcomes [94,95]. This approach and the aforementioned peptide grafting demonstrate once more the power of combining structural information with directed evolution and semi-rational design, in order to obtain capsids with altered tropism and/or immune evasion properties.

### 2.2. Integrating Features of Different Wild-Type Capsids into Engineered Progeny

The breadth of naturally occurring AAV variants [96,97,98,99] provides a solid basis for the development of a large portfolio of vector capsids with differing transduction profiles. To investigate the differences among the serotypes and to pinpoint the regions and amino acid residues that define each serotype, Vandenberghe et al. compared AAV isolates and identified singletons, i.e., divergent amino acid residues, among homologous variants [100]. This helped to define critical residues that affect vector yield and transduction efficiency, and to improve on such features by reversion of the respective singleton towards the conserved residue. The same approach was also applied to other parvoviruses, such as the human Bocavirus 1 (HBoV1) [101] and the minute virus of mice (MVM) [102], to study infectivity and the mechanical elasticity of the virus capsid, respectively. Following this train of thought, several groups reckoned that a reversion of variant amino acids between related AAV variants towards an ancestral version may yield capsids with novel and potentially beneficial features. Indeed, two studies from 2015 exemplified the potential of such an approach by using the rational design of putative ancestral capsids [103] or by employing directed evolution of an ancestral capsid library [104]. Both approaches produced capsids with increased thermostability and favorable transduction properties. One of these variants, namely, Anc80L65, that was generated by Zinn et al. [103], was later found to exhibit superior transduction efficiency upon local injection in the inner ear [50,105,106] or eye [107,108].

#### 2.2.1. Rational or Partially Randomized Integration of Residues from Multiple Serotypes through Domain and VR Swapping

Considering that the strongest variations between different AAV serotypes lie within their variable capsid regions, a straightforward way to interrogate the phenotypes exerted by different VRs is to swap these domains and to generate chimeric *cap* sequences. An initial example hereof was reported by Hauck and Xiao [109] who investigated the domains of AAV1 that infer its superior transduction of muscle tissue as compared to AAV2. By swapping domains between the AAV1 and AAV2 capsids, an amino-acid stretch between VP1 positions 350 and 423 was determined as essential for driving the high muscle transduction of AAV1. A similar approach was followed by Shen et al. [43] who swapped domains between AAV2 and AAV8 in order to identify those that are important for the superior murine liver transduction of AAV8. Assaying 27 chimeric domain swap capsids demonstrated the functional importance and interplay of VR IV and VIII domains (referred to as interstrand loop IV subloops 1 and 4 in the work by Shen et al.).

A more high-throughput approach was presented by Marsic et al., who generated a combinatorial VR library that incorporates variant amino acid residues from the VRs of different serotypes [46]. By first packaging single- or double-VR libraries and using the packaged DNA for PCR-based construction of a combined library with increased chances of viral packaging, this allowed the creation of an AAV2-based library with 156 permutated positions derived from the VRs of other serotypes. An in vivo selection in mouse liver then yielded capsids with enriched motifs in VR IV, V and VI, and an improved liver transduction as compared to their parental AAV2. A similar approach has recently been described by the same group for AAV3B, now changing the selection pressure by screening in 3D cultures of human hepatocytes in vitro [110]. This directed evolution approach led to the isolation of AAV3B-DE5, which differs in 24 amino acid residues from its parent AAV3B and exhibits improved transduction of human hepatocytes as well as reduced neutralization through NAbs in human sera.

Based on the same rationale, i.e., to employ structural information for creation of domain-swap variants as harnessed in the VR shuffling presented above, an algorithm-based prediction of beneficial recombination sites via the SCHEMA pipeline [111] has been introduced into the AAV field. Specifically, Ho et al. aimed to find optimal crossover positions in rationally designed chimeric capsids derived from AAV2 and AAV4 [112]. While disregarding functional importance, the SCHEMA algorithm employs structural information of intra- and inter-subunit interactions to calculate disruption scores of the resulting chimeras with one or two crossover sites between AAV2 and AAV4. In theory, crossover positions that result in (i) minimal disruption and (ii) maximal numbers of mutations as compared to the parental capsids would be ideal to create divergent but functional capsid libraries that promise success in downstream selection schemes. While Ho et al. [112] could not establish a correlation between genome packaging and disruption scores, an improved resistance to DNase-based degradation of packaged viral genomes was observed for capsids with lower theoretical disruption, proving the usefulness of SCHEMA to predict crossover sites that yield intact capsid derivates. This approach has recently been extended by Ojala et al. [44] for the prediction of seven optimal crossover sites of chimeric capsids derived from parental serotypes AAV2, −4, −5, −6, −8 and −9, creating a theoretical diversity of 1.7 million variants. After library production, the authors noted a de-selection of AAV4 and AAV5 blocks upon packaging, indicating a potential interference with the other serotype capsid stretches. In a subsequent Cre-dependent in vivo selection where enrichment was assayed by Cre-recombination of functionally transducing capsid variants, the SCHEMA library outcompeted other co-delivered libraries (based on error-prone PCR, random peptide display and DNA family shuffling). This screen yielded the lead candidate capsid SCH9 that robustly transduced neuronal stem cells in the subventricular zone upon intrasubventricular injection.

#### 2.2.2. DNA Family Shuffling for AAV Vector Evolution

In contrast to the aforementioned VR shuffling or SCHEMA approaches, DNA family shuffling harnesses the partial DNA sequence homology of at least 55% that exists between AAV *cap* genes of natural variants [113] and then shuffles them based on this homology in order to create a highly diverse library of capsid sequences. Head-started by three publications in 2008 [45,114,115], AAV DNA family shuffling has rapidly become a prominent tool for directed evolution of novel AAV capsids. In essence, this method relies on the digestion of AAV *cap* genes with DNase I and their subsequent reassembly over two consecutive PCRs. This creates libraries of “shuffled” *cap* variants either combining properties of the parental capsids or displaying entirely novel features for selection. Key considerations for this technique are (i) the choice of parental capsids and the use of codon-optimized variants thereof with higher DNA sequence homology [45,113], (ii) the incubation conditions with DNase I that define the average length of fragments for subsequent re-assembly [116], and, most importantly, (iii) the selection pressures employed during directed evolution [53]. Many of these shuffled capsid variants gain fundamentally different functional and immunogenic profiles as compared to their individual parental capsids. Hence, it is possible to extend the transduction-based selection procedure (i.e., iterative amplification in on-target cells or organs) by adding an additional selection pressure imposed by immunoglobulins comprising anti-AAV antibodies. This step enriches capsids with reduced antibody recognition, which may also be less susceptible to NAbs and hence allows for an immune escape in patients carrying these antibodies [45]. Consequently, shuffling can generate capsids that exhibit highly useful features such as immune evasion, enhanced transduction and retargeting towards a tissue of choice as defined by the selection process. Such selections allow for the enrichment of variants that transduce cell lines which are poorly permissive to the parental serotypes, as was demonstrated, for instance, by Maguire et al. for human glioblastoma cells [117]. Combining selection in liver cells with an immunoglobulin-based depletion, the AAV-DJ capsid was selected from a shuffled library as well [45]. This chimera of AAV serotypes 2, 8 and 9 is capable of transducing a broad range of cell types with high efficiency in vitro, exhibits strong murine hepatic transduction in vivo, and presents an excellent scaffold for peptide display, as exemplified by the successful selection of derivatives for intranasal delivery. As mentioned above, AAV-DJ has also been employed as a scaffold to create capsids for transduction of cells in the murine inner ear [41,91].

Similar to the results obtained by directed evolution with AAV peptide display, in vivo retargeting of AAV capsids can also be achieved by screening of shuffled libraries in animal models. This was, for instance, demonstrated by in vivo selection of shuffled AAV libraries for transduction of the murine heart [118] or CNS [119]. AAV-LK03 [120], on the other hand, was identified from a shuffled library that was screened in vivo for liver transduction in mice with humanized livers. This capsid, which is derived mostly from AAV3B, exhibits a strong transduction of human but not mouse hepatocytes, making it an ideal vector for liver-directed gene therapy in humans [121]. Towards the same aim, Paulk et al. also screened a shuffled library in a xenograft humanized mouse model (hFRG) and additionally included negative selection through pooled human immunoglobulins. This yielded AAV-NP59, a capsid with a strong tropism for human hepatocytes and lower NAb binding than LK03 [122].

As demonstrated by these examples, AAV libraries created by DNA family shuffling can be subjected to different forms of enrichment procedures in vitro or in vivo. In order to analyze the library composition and sequences of enriched candidates, different forms of sequence interrogation can be employed. While the classical approach of Sanger sequencing is practically limited to a low number of sequenced clones (in the range of 10^1^–10^2^), different NGS approaches allow for a more thorough examination of library composition and selective enrichment. PacBio’s SMRT sequencing permits long-read sequencing covering the whole *cap* gene at a depth of at least 10^4^ reads [122]. While this can help in identifying the most enriched variants, it does not necessarily grant the ability to track individual variants with a low relative abundance in initial viral libraries with a typical diversity of 10^6^ or more variants. In contrast, the latter is more readily possible in peptide-display or barcoded screens. Here, high read numbers (>10^7^ reads) can be acquired using Illumina-based sequencing, as the readout requires only the interrogation of a small stretch of DNA instead of the whole *cap* gene [24,68,71]. In an effort to combine the best of both (sequencing) worlds and to apply this short-read sequencing strategy for enrichment analyses of shuffled AAV libraries, a workaround was created by Pekrun and colleagues [123]. Instead of reading the *cap* sequence itself, the authors added a highly diverse barcode pool downstream of the *cap* gene to enable Illumina-based tracking of variant enrichment. The exact *cap* sequences were then identified in a secondary step, where an enriched barcode sequence (as identified by Illumina NGS) was used as primer-binding site, permitting the PCR-based amplification and subsequent Sanger sequencing of the respective variant. In a follow-up study by de Alencastro et al. [124], the same barcoding approach was employed to interrogate general parameters of AAV library selection schemes. This demonstrated a higher reproducibility for screens where transduction was performed with higher multiplicity of infection as well as the appearance of competition-based artifacts that do not translate to single-variant behavior. Similar interrogations may prove highly beneficial in future AAV shuffling screens, as they allow for the observation of variant enrichment under different selection conditions.

In the aforementioned study of Pekrun et al., screening of a shuffled capsid library was performed in vitro for the transduction of human pancreatic β-cells [123]. Upon systemic examination in liver-xenografted mice, the lead capsid, AAV-KP1, was found to also transduce both human and mouse hepatocytes with great efficiency. As AAV-KP1 has 93% identity to AAV3B, Cabanes-Creus et al. studied why AAV-KP1 possesses this property, while AAV3B and, for instance AAV-LK03, are somewhat restricted to transduction of human hepatocytes [125]. By swapping domains from AAV-KP1 to AAV3B, the authors could pinpoint the transduction phenotype for mouse hepatocytes to a single amino acid in AAV-KP1, namely, the insertion of threonine at VP1 position 265. Removing this threonine in AAV-KP1, or adding it to AAV3B or AAV-LK03, allowed the authors to disable or enable the transduction of murine vs. human hepatocytes in a hFRG xenograft mouse model, respectively. The 265T insertion may therefore allow for the pre-clinical study of human-tropic AAVs in a murine setting.

#### 2.2.3. Combinatorial Shuffling and Domain Swapping

Finally, we highlight combined approaches comprising DNA family shuffling and subsequent domain swapping. In a recent study, Cabanes-Creus et al. screened an AAV1- to 12-based shuffled library in livers of hFRG mice [126]. AAV-SYD12, one of their lead candidates, outperformed all other benchmark capsids in the transduction of human hepatocytes from 17 different donors in these mice. To dissect the molecular determinants of the AAV-SYD12 phenotype, the authors applied domain swapping by grafting VRs from AAV-SYD12 onto AAV8. Next, an analysis of transduction efficiency was performed in mice with low or high degrees of replacement of murine with human hepatocytes. This strategy enabled highly informative insights into the synergistic effects of the VRs from different origins, and identified several VRs as critical for an enhanced uptake into human hepatocytes in vivo (VR I from AAV2 as well as VRs VII and VIII from AAV7). Moreover, other VRs were found to be critical for enhancing functional transduction after cellular uptake (VR I from AAV8, IV from AAV10, and VII from AAV7). This, in turn, allowed for the rational design of AAV7- and AAV8-derived capsids displaying the enhanced-uptake phenotype by merely engrafting the respective VRs, and thus demonstrates the superb synergism that can be generated by combining AAV DNA family shuffling with systematic domain swapping for subsequent functional analysis. Another pivotal example of combinatorial DNA family shuffling and following functional dissection was presented in work by Albright et al., where shuffling between AAV1 and AAVrh.10 was employed in order to identify residues from AAVrh.10 that enable crossing of the BBB [127]. By studying several shuffled capsid variants, eight amino-acid residues in VR I were found to be sufficient to introduce the BBB-crossing phenotype from AAVrh.10 into AAV1, yielding capsid AAV1RX. Interestingly, the introduction of these eight residues also detargeted AAV1RX from the liver, which is a common off-target that is strongly transduced by both of its parental serotypes.

In summary, data presented by multiple research labs around the globe have clearly demonstrated the vast potential of high-throughput methods for AAV capsid engineering. The following dissection of phenotypic determinants using domain swapping approaches not only helped to uncover genotype-phenotype associations, but also facilitated the semi-rational design of new AAV capsid variants. Combined with the findings on capsid structures, exposed epitopes, functional analysis and cell-surface receptor expression, this now offers intriguing options for future improvements, as further discussed below.

## 3. New Synthetic Biology-Inspired Approaches

**Figure 2:**
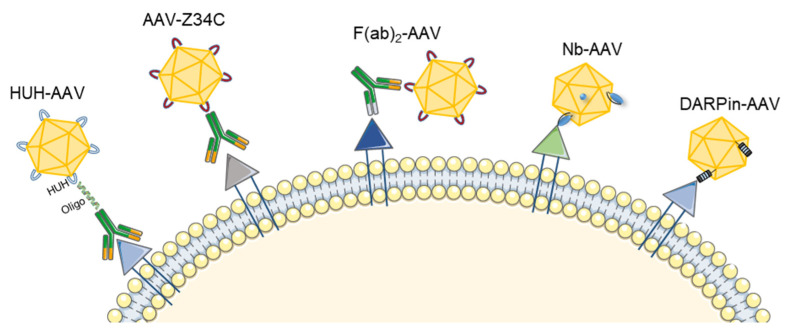
Synthetic biology-inspired approaches to modify AAV vector tropism. Antibodies can be coupled to AAV capsids via a covalent interaction between a HUH tag in the AAV capsid protein and the antibody, which is enabled through an oligonucleotide bridge (HUH-AAV) [32]. Non-covalent interactions can also be harnessed, for instance, by using an Fc-binding Z34C domain integrated into the AAV capsid (AAV-Z34C) [135] or a bispecific antibody that recognizes a conformational epitope [136] or a tag inserted into the AAV capsid (F(ab)_2_-AAV) [137]. Other molecules such as nanobodies (Nb) inserted into the GH2/GH3 loop of VP1 [29] or DARPins integrated into the same loop [30], fused to the VP2 N-terminus [138,139] or covalently linked [140] can also be used to efficiently retarget AAV vectors. This figure contains free clipart from https://smart.servier.com/ (accessed on 1 April 2022).

As briefly discussed before in the context of peptide display and domain swapping, rational design has become a prime tool and a valuable add-on to complex screening and stratification-based technologies. Important to mention is that directed engineering approaches extend beyond genetic modifications or engraftment of heterologous entities. As these become an intrinsic property of the capsid itself, they are often limited in sequence and size. Non-genetic or mixed approaches, in contrast, allow one to expand the range of molecules or ligands that can be coupled to AAV vectors. To this end, a multitude of chemical and biochemical approaches exist to couple oligonucleotides [128], sugar moieties [129,130,131], proteins [132], or synthetic polymers such as polyethylene glycol [133,134] to the AAV capsid. Despite their undisputed promise, many of these concepts are still in the early stages of proof-of-principle studies. Thus, due to space limitations, we will not elaborate further on all these different concepts and instead refer the reader to the above-mentioned original literature. In the following, we focus on two recent leaps in AAV retargeting using high-affinity binders either based on antibodies, nanobodies (Nb) or designed ankyrin repeat proteins (DARPins) (for an overview see Figure 2).

### 3.1. Antibody-Mediated AAV Retargeting

Monoclonal antibodies have emerged as a rapidly growing class of therapeutic agents with a wide range of indications and many approved products, especially for oncological and immunological diseases [141]. The idea of harnessing high-affinity antibodies to retarget AAV vectors dates back twenty-four years to work from Yang and colleagues [142]. At that point, many studies were fueled by the need for AAV vectors with broader tropisms, especially to deliver cargos to transduction-resistant cells, such as those from the hematopoietic lineage. However, the incorporation of large ligands into the AAV capsids was found to be challenging, as this often interferes with capsid assembly, genomic titer yield and/or infectivity [135,142,143,144]. Therefore, most research work focused on either piggybacking antibodies to the AAV surface or genetically incorporating binders or smaller versions of antibodies.

#### 3.1.1. Genetic Fusion of Single-Chain Variable Fragments (scFv) and Ligands to the AAV Capsid

In a study by Yang et al., the variable domain of a scFv targeting CD34 was fused to the N-terminus of the AAV2 VP2 protein [142]. The resulting vectors showed a superior transduction of CD34^+^ cells by more than 100-fold as compared to the underlying WT AAV2 but achieved only low titers of >2 × 10^2^ transducing units per ml. Notably, the authors had to supply WT AAV2 VP2 proteins to achieve virus assembly, which is disadvantageous as WT capsids would interfere with the retargeting strategy. An interesting notion in this work was that only a fusion to the VP2 protein allowed retargeting to KG-1 cells (an acute myelogenous leukemia cell line positive for CD34), whereas VP1 or VP3 fusions were not well tolerated. This feature of VP2 was later harnessed by multiple labs to insert a variety of ligands, e.g., a serpin-receptor targeting epitope (KFNKPFVFLI) [62], the fractalkine chemokine domain (FKN) and the human hormone leptin (LEP) ligands [144], or even fluorescent proteins [144]. The insertion of the latter, however, affected the genomic titer (one–two logs lower than WT) and particle infectivity (three to four logs drop) [144], hinting that insertions exceeding 18 kDa are not well tolerated. Follow-up studies later showed that the choice of promoters, the plasmid design and the ratios of different components (i.e., VP1, VP2-fusion protein, VP2 WT and VP3) affect packaging efficiency and vector potency. For instance, Lux et al. used a GFP-VP2 N-terminal fusion to generate fluorescent AAV particles that were comparable to WT AAV2 in titer and infectivity [145]. In addition, Asokan et al. fused Gaussia luciferase, a 19 kDa protein, to the N-terminus of VP2 without affecting titer or transduction efficiency of the resulting vectors. This enabled the tracking of bioluminescent viral shells based on AAV1, AAV2 and AAV8 in vivo [146]. Finally, recent work from the Buchholz lab showed successful fusion of DARPins to the N-terminus of VP2 that was used to generate cancer-targeting AAV vectors [138] (as further discussed below).

#### 3.1.2. Use of Bispecific Antibodies as Bridging Molecules

Bispecific antibodies are engineered molecules that were designed to bind two distinct antigens at the same time and that are commonly employed for T-cell redirection and engagement [147]. Bartlett and colleagues [136] sought to harness their unique property to retarget AAV vectors. To this end, a bispecific F(ab’gamma)2 antibody was used that is composed of two Fab arms, one targeting αIIbβ3 integrin (on the cell surface) and the other with specificity towards the AAV2 capsid (derived from the A20 antibody that specifically binds assembled AAV2 particles) [148]. As the natural ligand of the αIIbβ3 integrin receptor, fibrinogen, is endocytosed upon binding to the receptor, the authors hypothesized that the antibody-conjugated AAV will be internalized as well. Indeed, the AAV2-antibody complex mediated transduction of DAMI and MO7e human megakaryoblast cells that express αIIbβ3 integrin and are refractory to natural AAV2 transduction. However, the transduction efficiency was over 10-fold lower than that observed in permissive cell lines (such as HeLa cells). Notably, the AAV vectors covered with antibodies did not transduce the off-target HeLa cells and hence seemed to mediate more specific transduction. As the natural AAV2 binding site to heparan sulfate proteoglycan (HSPG) was still intact, it remains unclear whether the lack of αIIbβ3 integrin on the HeLa cells has contributed to the apparent specificity or whether the steric hindrance posed by the antibody has prevented the particles from interacting with their nascent receptor.

#### 3.1.3. Generation of Universal Templates Based on Antibody Binding Domains

As it remains challenging and time-consuming to establish bispecific antibodies for each receptor and AAV serotype, Ried et al. [135] aimed at generating a universal AAV targeting construct that allows for the coupling of any antibody via its Fc part. Specifically, a minimized immunoglobulin G (IgG) binding domain of protein A (Z34C; a 34 amino-acid two-helix domain) was first inserted into VP1 position 587 of AAV2. The resulting vectors could be packaged efficiently but gave around 10- to 20-fold lower genomic titers. Next, various IgG molecules targeting CD117, CD29 or CXCR4 were coupled to the capsid surface via their Fc region. As observed before by Bartlett et al. [136], the transduction of target cells with these AAV-antibody mixtures was more specific as compared to WT AAV2, but less efficient. Gigout and colleagues [149] aimed to improve this system by incorporating the Z34C fragment into only a portion of the capsid proteins. This could be achieved by supplying WT capsid proteins in *trans* during the production process thereby creating mosaic vectors that contain different ratios of WT and Z34C VP proteins. The authors showed that the transducing titers of the resulting vectors negatively correlated with an increased ratio of Z34C proteins, hinting towards a deleterious effect of the Z34C insertion if present in all capsid proteins. Next, the transduction efficiency of the mosaic vectors was tested in the presence and absence of targeting antibodies against CD117 or CD29. Here, the vectors with 25% Z34C-VP content performed the best and even outperformed AAV2 WT with 11- to 18-fold higher transduction abilities.

Almost two decades later, Kuklik et al. [137] reported an approach combining the two above-mentioned strategies [135,136]. Instead of relying on A20 antibody binding [135], which only recognize AAV2 [148], a peptide epitope (2E3) derived from the proprotein-convertase subtilisin/kexin type 9 (PCSK9) was inserted into different regions of the AAV2 surface. Then, bispecific antibodies were designed that target both, the 2E3 epitope in the AAV capsid and a target receptor on cells, thereby bridging the AAV-target receptor interaction. One of the most promising constructs in this work, rAAV-2E3.v6, in which the 2E3 epitope substituted the AAV2 capsid residues 581 to 589, gave titers comparable to AAV2 WT and efficiently and specifically transduced target cells expressing the target receptors FAP or PD-L1.

Taken together, antibody-mediated approaches have provided solid in vitro evidence for the possibility to redirect AAV vectors to cells expressing a target receptor. However, the in vivo stability of the AAV-antibody complexes is still a key concern for future applications. To overcome this challenge, Ponnazhagan et al. constructed a system that relies on the high-affinity avidin-biotin linkage (Kd = 10–15 M), which is 10^3^ to 10^6^ times higher than a standard antibody-antigen interaction [150]. In more detail, the AAV capsids were first biotinylated in vitro and then incubated with a streptavidin-coupled ligand targeting epidermal growth factor receptor (EGFR) or fibroblast growth factor receptor 1α (FGFR1α). These vectors showed a more than 100-fold improvement in their transduction efficiency as compared to the WT AAV2 control. Notably, AAV capsids can also be biotinylated in a site-specific manner by inserting a 15-amino acid biotin acceptor peptide (BAP). An enzyme from *Escherichia coli* (BirA biotin ligase) is then used to ligate the biotin to the acceptor peptide [151,152]. Although biotin-streptavidin-based therapeutics possess a great potential, it is important to note that the immunogenicity of streptavidin [153] and its broad non-specific binding are of concern [154].

#### 3.1.4. Covalent Binding of Antibodies to the AAV Capsid Surface

Covalent bonds are the strongest and most stable chemical bonds found in nature. Therefore, using covalent interactions to link antibodies or other targeting molecules to the AAV capsid surface might represent an elegant alternative to the previously mentioned non-covalent strategies. This can be achieved by either randomly attaching the targeting molecule [155] or by using defined areas on the capsid surface. In the following, we will focus on the latter strategy as random conjugation methods often impacted vector titer or functionality [131,156]. So far, several research groups have established methods to covalently link antibodies to specific sites in the AAV capsid [32,130,140,157]. We apologize to colleagues whose relevant work we cannot highlight in the following due to space reasons and refer the reader to the aforementioned literature.

Zdechlik et al. inserted a mMobA HUH tag (10–30 kDA) into the VR IV of either VP1, VP2 or VP3 of AAV-DJ [32]. This tag can form covalent bonds with ssDNA-conjugated antibodies [158]. Insertion in VP3 was not well tolerated and resulted in a sharp decrease in titers, whereas incorporation into VP1 and VP2 was possible. The authors then focused on the VP2 incorporation of the tag and showed that a conjugation of antibodies was, in principle, successful and retained the infectivity of the vectors. They could also demonstrate the specificity of these vectors in a variety of cell lines (Jurkat, U-251 MG), primary cells (primary neuron hippocampal neurons) and mixtures of on- and off-target cells (anti-LICAM-AAV to target neurons in a mixture with glia cells). While this concept is interesting for platform development, as the “template” vector has to be produced only once, several process optimizations are still required. For example, the low incorporation of the VP2-tag into the AAV particles has to be addressed, as this is a critical determinant for the success of conjugation and defines the number of antibodies displayed per viral capsid. In addition, the efficiency of the conjugation reaction itself also remains unclear, as a purification of the fully conjugated AAV (VP2-HUH-antibody) products was not performed.

Yet another system was described by Muik et al. that allows one to covalently link scFvs (single-chain variable fragments) and DARPins to a universal AAV template [140]. The authors utilized a protein-*trans-*splicing (PTS) approach mediated by intein domains derived from DNA polymerase III (DnaE) of *Nostoc punctiforme* (Npu). To this end, the C-terminal domain of Npu DnaE was fused to the N-terminus of the AAV2 VP2 capsid protein. In a second step, the targeting domains (scFvs or DARPins) were fused to the N-terminus of Npu. The NpuC-AAV and the targeting-NpuN fusion proteins were purified separately using density gradient centrifugation or affinity chromatography, respectively. Finally, both components were combined in vitro in a splicing buffer that mediates the protein-*trans-*splicing reaction. The final vectors showed high selectivity for their target cells (comparable to genetically fused targeting domains), but with surprisingly lower off-targeting rates. As observed by Zdechlik et al. [32], the coupling to the NpuC-VP2 protein was rather inefficient and reached only 15% under the best reaction conditions, which still has to be optimized before this strategy can be transferred to a clinical setting that requires high doses.

With their high degree of modularity and the ability to incorporate different substrates, from small peptides to scFVs or complete oligo-tagged antibodies, both the HUH-tag- and NpuC/N-based systems could significantly expand the targeting range of AAVs. A limitation, however, is that both strategies require laborious production and monitoring of each component, followed by a conjugation reaction that introduces high variability.

### 3.2. DARPin-Mediated Viral Vector Retargeting

Proteins that contain Ankyrin repeats (AR) are very abundant in nature and are especially found in eukaryotes. An AR motif consists of 30–34 amino acids and folds in a unique helix-turn-helix conformation. Various numbers of repeated modules are then arranged to form linear structures that mediate specific protein-protein interactions [159]. The reported binding affinities of these AR proteins in nature is in the nanomolar range, which resembles therapeutic antibodies and hence inspired the engineering of these molecules for biomedical applications [160,161]. In 2004, the Plückthun lab reported the first successful construction of DARPins designed to bind the maltose binding protein of *Escherichia coli* and eukaryotic protein kinases with high affinity and specificity [162]. Soon it was recognized that combining DARPins with viral vectors, e.g., lentivirus [30,163] or AAVs [30,138,139,164,165], could dramatically expand the range of applications, as this allows for the specific delivery of nucleic acids. Still, the challenges in translating such approaches to viral vectors are manifold, with the two largest hurdles perhaps being (i) the exposure of stably folded domains on the capsid surface that allows for a correct interaction with the receptor, and (ii) the interference of large insertions with viral capsid formation.

In 2011, the Buchholz lab demonstrated the first insertion of DARPins into lentiviral vectors, which allowed targeting of HER2/neu-positive tumors [163]. Follow-up work from the same lab showed that the concept could be transferred to AAV [138,139]. In contrast to lentiviruses that have a diffuse glycolipid coat, AAV is a non-enveloped virus with a rigid protein shell, which necessitates a careful assessment of amenable insertion sites. Specifically, the N-terminus of VP2 was exploited, a region previously reported to allow the insertion of foreign proteins [144,145,146]. To enable the formation of infectious viral particles, the VP1 and VP3 proteins remained in their WT configuration and were added *in trans*. To ablate the natural AAV2 tropism and de-target the capsid from its natural target cells, mutations were introduced to the HSPG-binding site in all VP proteins. Remarkably, all AAVs tagged with DARPins could be produced at titers comparable to the WT AAV2 vector controls with only minor reductions depending on the DARPin [139]. In this study, three different DARPins were evaluated, i.e., one Her2/neu-specific DARPin (DARPin-9.29) [138] and two others binding to the surface receptors CD4 (DARPin 55.2 [160]) or EpCAM [161]. All DARPin-targeted AAVs, namely, AAV-Her2, AAV-CD4 and AAV-EpCAM, showed high levels of on-target activity and no detectable off-targeting. In brief, after systemic administration in xenografted mice, AAV-Her2 detected 75.7% of tumor foci and AAV-CD4 targeted 4.4% of all human CD4^+^ lymphocytes. Likewise, AAV- EpCAM targeted more than 90% of EpCAM-positive tumor cells in a mixture of cells, even when these cells were under-represented in the mixture. To subsequently assess the promise of the tumor-targeted AAV-Her2 vectors to combat Her2/neu-positive tumors, the cytotoxic gene herpes simplex virus (HSV) *thymidine kinase* was packaged into the engineered viral capsid. When injected into mice harboring Her2-positive tumors, the AAV-Her2 efficiently targeted Her2^+^ cells and resulted in a more effective reduction in tumor mass than the clinical antibody control (Herceptin).

Hepatotoxicity is considered a dangerous side effect of AAV vector administration, especially at high doses [166,167,168]. One observation in prior work by Münch et al. was that both, WT AAV2 and AAV-Her2, could target tumor tissue at high efficiencies. Importantly, however, AAV-Her2 showed improved specificity that alleviated liver toxicity [138]. In yet another recent study from Stone et al., the 55.2 DARPin was fused to the N-terminus of AAV6 VP2 to target CD4^+^ blood cells in immunocompetent rhesus macaques [169]. Interestingly, the biodistribution of AAV6 -CD4 did not significantly differ from the parental AAV6 and no transgene expression was detected in blood cells or in any organ. Importantly, in contrast to the study by Münch and colleagues [139], the AAV6-CD4 conjugation products were not enriched by iMac, which has been shown to result in significantly lower transduction efficiencies. In addition, with 5.6%, the in vitro transduction of macaque CD4^+^ cells was very inefficient as compared to the one observed in cells of human origin (~39%). This highlights the importance of prior in vitro screening for efficient DARPin-AAV conjugates and the subsequent purification and enrichment steps.

Besides traditional cytotoxic genes, immune checkpoint inhibitors (ICIs) represent a novel class of immunotherapy drugs that have revolutionized cancer therapy. While impressive results were reported with these agents, the unspecific expression of ICIs and the resulting immune-associated adverse events still represent a challenge in their application. Reul and colleagues aimed at overcoming this challenge by harnessing tumor-specific AAV-DARPin vectors. To this end, an anti-(α)PD-1 construct (programmed cell death protein 1) was packaged into the Her2-AAV vector and injected into mice with subcutaneous RENCA-Her2/neu tumors [164]. In line with the study from Münch et al. [138], both AAV2 and AAV-Her2 led to comparative αPD-1 expression levels in the tumor tissue. Importantly, however, AAV-Her2 showed improved specificity. Regarding therapeutic efficiency, tumor growth inhibition in the AAV-Her2 mice cohort was modest and only detected in combination with adjuvant therapy (chemotherapy). Consequently, a minor survival advantage was observed, which necessitates further engineering and optimization of transgene cassettes and/or doses. Moreover, a separation of AAV particles containing the desired DARPin fusion from unwanted VP2-deficient particles may further potentiate transgene expression, as mentioned before [139].

In the context of cancer treatment, it is important to mention that autonomous parvoviruses (APV) have also been employed to target a wide range of cancer types [1]. These vectors were mostly derived from two rodent parvoviruses, namely, the H-1 parvovirus and MVM. In contrast to AAV, where the complete viral genome is substituted by a transgene of interest, APV vectors are composed of a wild-type genome with an intrinsic propensity to replicate in tumor tissue [170]. The APV then induces tumor remission by two complementary mechanisms: (i) direct lysis of cells and (ii) stimulation of the immune response. As shown for AAV vectors, the combination of APVs with other adjunct therapies, such as small molecules, chemotherapy, or immunotherapy, further potentiates their anti-tumor activity [171]. Nonetheless, the lack of specificity and the low in vivo efficiency of these APVs have restricted their application to selected clinical trials (NCT01301430, NCT02653313). In view of the recent efforts to target AAVs to tumor tissue, it could be rewarding to directly transfer these strategies to APV or to make use of hybrid viral vectors that potentially combine the assets of vectors derived from different viruses [172].

Finally, in addition to the successes with DARPin-armed AAVs in cancer research, two recent studies are noteworthy that aimed to further expand their applications to other targets. By inserting a murine CD8-specific DARPin into the GH2/3 loop of AAV2 VP1, an AAV-mCD8 vector was generated that targeted CD8^+^ cells in whole murine splenocytes with high efficiency (26-fold higher than unmodified AAV2) and >99% specificity [30]. Moreover, in a study by Hartmann and co-workers, interneurons were targeted by a GluA4-specific DARPin [165].

### 3.3. Nanobody-Mediated Targeting of AAV Gene Therapy Vectors

Nanobodies (Nb) are derived from the VHH domain of heavy-chain antibodies that naturally occur in camelids or sharks. These antibodies differ from those in humans in that they only contain heavy but no light chains [173]. Importantly, Nbs are engineered versions that are composed of one variable chain, i.e., a fraction of these antibodies. Thus, with roughly 15 kDa, they are very small in size as compared to conventional antibodies with ~150 kDa. The small size of Nbs is often considered as an advantage, as they can better penetrate tissues and diffuse to their site of action [174]. In addition, this size seems to perfectly lie in the optimal range for insertion into AAV capsids [175]. The Koch-Nolte lab was the first to fit cell membrane protein-specific Nbs into the GH2/GH3 loop of the AAV2 VP1 protein [29]. Three different Nbs directed against CD38, ARTC2.2 or P2X7 were displayed on the AAV2 capsid and re-directed AAV2 to HEK293 cells expressing the respective target receptor. Importantly, targeting P2X7 allowed a higher degree of specificity (500-fold) than ARTC2 (10-fold), which highlights the need to first screen for multiple targets and Nbs to achieve the best on-to-off target ratio. Next, the AAV2 VP1-conjugated Nb was combined with the VP2/VP3 proteins from other AAV serotypes, namely, AAV8, AAV9 and an AAV1 with a peptide insertion [49]. Using this approach, the authors could target cells that were untargetable with the AAV2-Nb conjugate. For example, all mosaic vectors transduced >70% of Yac-1 cells (a murine lymphoma cell line), whereas the AAV2-Nb could only transduce 5%. This reflects the importance of other steps in the transduction pathway beyond binding to the primary cell surface receptors, such as the binding of co-receptors, intracellular trafficking and uncoating. In another study, Hamann et al. followed a similar strategy as conducted with DARPins before [138,139], by fusing Nbs to the N-terminus of VP2 [176]. This was combined with mutations in surface-exposed tyrosine residues that have been shown previously to enhance transduction [177]. Interestingly, incorporation into VP1 (VP1-Nb) or fusion to VP2 (VP2-Nb) resulted in vectors with comparable efficiencies, albeit a higher specificity was observed with VP1-Nb (up to 199-fold as compared to only 15-fold for VP2-Nb). This, however, can be attributed to the fact that WT VP2 (without nanobody fusion) was supplied *in trans* during vector production, as in the original work by Yang et al. [142], which would likely compete with VP2-Nb for incorporation into the viral particles. The authors also aimed at expanding their AAV-Nb toolbox to bispecific Nbs with known higher affinity for the target. Unexpectedly, incorporation of these molecules into VP1 resulted in lower targeting efficiency than with the monovalent Nb. This could be explained by the larger size of the bispecific Nb that exceeds the limits of the GH2/GH3 loop and/or steric hindrance caused by the mere presence of the Nb, which may slow down or block the transduction of cells.

Taken together, redirection of AAVs through the incorporation of Nbs is a promising, rapidly evolving technology that holds great potential for future targeted applications. As for the previously mentioned DARPin-based approaches, a current limitation remains the laborious screening for new DARPins and Nbs that efficiently bind to a receptor of interest. A direct translation into AAVs is also often not possible, and extra screening rounds are usually necessary to test for both, efficient incorporation and functionality. Finally, the establishment of a one-fits-all purification platform for all the different AAV-Nb or DARPin products, such as POROS CaptureSelect AAVX Affinity resins [178,179] or AVB Sepharose [180], is highly desirable but may be challenging.

## 4. Data-Driven Capsid and Library Design

While rational engineering approaches can yield highly functional capsids with good on-target precision (as demonstrated by Nb- and DARPin-AAV fusions), the throughput is very low, and the design process is tedious. Directed evolution, on the other hand, employs large capsid libraries that can be interrogated in a high-throughput manner, and can therefore explore a much greater sequence space. However, a high degree of non-functional variants is usually generated as an unwanted side product [53]. Thus, to boost the chances of identifying functional offspring within a complex capsid library, one can integrate data-driven knowledge during library creation to minimize the impact of defective sequences on library size and vitality. One example thereof that has been noted before has been reported by Davidsson et al. [92], who performed peptide display using sequences derived from proteins that were known to exhibit the desired phenotype of retrograde axonal transport, rather than using randomized peptide display. This approach was supported by recent improvements of two essential technologies, i.e., advanced possibilities of synthesizing large pools of oligonucleotides that were designed by a computer algorithm [92], and NGS-based interrogation of capsid function through barcoding of capsid variants [68]. Illumina-based NGS allows for the deep monitoring of randomized short sequences, typically small stretches of the *cap* gene, displayed peptides or DNA barcodes that are linked to a given capsid variant. This is useful for monitoring of variant enrichment in on- and off-target organs [71], and it is especially useful in the context of barcoded screens for tracing of the fitness of a given capsid [68,76]. In the study of Ogden et al. [37], these technologies were applied on an unprecedented scale, as DNA barcoding was used to trace the viability of a comprehensive mutagenesis library, i.e., AAV2-based capsid variants with all possible single synonymous codon substitutions, amino acid substitutions, insertions, and deletions at each of the 735 residues. Each of these variants was tagged with a DNA barcode present on the viral genome to enable NGS-based tracking of capsid variants. Barcode sequencing of both, the plasmid library as well as the packaged viral genomes, then allowed for a quantification of the ability of each capsid sequence to assemble into a functional virion and thereby for an interrogation of the fitness over the entire single-mutant capsid landscape. This valuable dataset not only helped to identify residues that tolerate mutations (mostly within the VRs) and to determine which amino acids were more favorable, but it also enabled the detection of clusters of mutations that govern the in vivo transduction of different mouse tissues. Finally, it even facilitated the identification of an AAV gene embedded in an alternative open reading frame (ORF) overlapping with the *cap* ORF, encoding a so-far unknown protein termed membrane-associated accessory protein (MAAP). Injecting the single-mutant library into mice allowed for the classification of sequence clusters involved in the transduction of different tissues. Focusing on capsid positions between 561 and 588 (VP1 numbering), Ogden et al. then utilized the single-mutant dataset to create an additive model algorithm to design potentially viable multi-mutant capsids. While randomly chosen multi-mutants were mostly non-functional, the machine-guided variants derived from the additive model showed enhanced viability for up to 10 mutations. However, to predict multi-mutants with even larger distance towards the parental capsids, and to achieve higher accuracy of predicting viable capsids, both the available datasets and the employed algorithms would require upscaling.

### 4.1. Machine Learning in AAV Library Generation

Over the last years, machine learning (ML) has found its way into biology and biomedical research, and has enabled the meaningful interrogation of comprehensive datasets. Successful examples are found for many different biological applications such as image analysis, genetics/genomics and drug discovery [181,182,183]. By training ML algorithms with annotated datasets, these models can find features within these “training” data and thereby learn how to correctly predict their classification. A recent noteworthy example has been provided by Jumper et al. [184] with the prediction of 3D protein structures. As experimental determination of protein structure is tedious, in silico structure predictions based on physicochemical interactions or evolutionary relation have gained interest but remained mostly inaccurate. By employing the neural network AlphaFold, which was trained on protein structures and sequences available on the Protein Data Bank, Jumper et al. were now able to precisely predict protein structures based on an amino-acid sequence input and multiple sequence alignments of homologous proteins. Applying this model to the human proteome enabled confident structure predictions for a large number of human proteins [185]. Apart from structure prediction, another promising application for ML is the prediction of sequence-to-function relationships in protein engineering. Here, the goal is to use an input of unknown protein sequences and accurately predict their respective function [186]. This is especially promising for AAV vector design, as large parts of the AAV capsid sequence space remain unexplored and most vectors are flawed regarding either their elicited immune response, suboptimal transgene delivery, or inadequate vector yield. However, ML-based protein engineering brings great challenges as well. Choices of model design, data representation and training data generation have major impacts on the predictive power of such algorithms. Recent publications have now demonstrated that ML can be employed for AAV vector and library design as well, transforming the approach with which AAV libraries are designed and interrogated. The results, thus far, are mostly encouraging and lay an excellent foundation for continued research.

Pioneering work in this regard has been presented by Bryant et al. [187]. With the goal to replace random mutagenesis with a purely data-driven approach, the mutation landscape of AAV2 within capsid positions 561–588 was revisited from the Ogden study [37]. As random multi-mutants are largely unviable, directed evolution based on random diversification is incapable of accessing large parts of the multi-mutant sequence space due to an oversaturation of the practically limited library size with unviable variants. Hence, Bryant and colleagues aimed to use ML to explore this largely inaccessible sequence space. This was achieved by firstly testing different training datasets and ML algorithms for finding suitable combinations. Specifically, the authors tested three different ensembles of training data libraries, containing combinations of (i) a complete single mutation set plus randomly chosen double mutants, (ii) a complete single mutation set plus random multi-mutants with two–ten mutations distance towards the parental capsids, and (iii) random multi-mutants plus variants predicted from a baseline additive model. The capsids within each of these training sets were then synthesized and assayed for successful assembly and packaging of viral genomes, as was conducted before by Ogden et al. [37]. Using the three datasets for training three different ML algorithms (logistic regression, convolutional neural networks or recurrent neural networks) enabled a model-based selection of potentially viable multi-mutants from a given input set of seed sequences. Subsequently, each model was employed for the model-guided design of viable variants by iterative ranking and in silico mutation of multi-mutant sequences. Testing both model-selected and model-designed sequences with 5–29 mutations as compared to WT AAV2 demonstrated the success of predicting viable sequences. It concurrently validated the ability of machine-guided generation of diverse viable multi-mutants, which greatly outperformed random mutant capsids and variants designed by the additive baseline model. During model evaluation, a tradeoff between precision (i.e., the ability to correctly predict viable variants) and diversity became apparent. This tradeoff was best solved by neural network architectures, which enabled the creation of viable libraries that still exhibit deep sequence diversity.

Following a similar train of thought, Marques et al. also aimed to improve the viability of multi-mutant libraries [188]. Based on their previous work on virtual family shuffling [46], Marques employed an AAV2 library that contained the previously identified motif D_492_G_493_E_494_-D_499_F_500_ (within VR V), 33 degenerate positions within variable regions and known antibody- and proteasome-evading residues. NGS-based tracking of variant assembly into functional capsids from this virus library was used to train either neural network or support vector machine algorithms. Achieving 72% accuracy in predicting viable candidates, the authors used their trained model for in silico testing of a single WT residue assay. Therefore, 33 theoretical libraries were generated and assessed in silico where 32 out of the 33 degenerate residues were diversified, while the remaining WT residue was maintained at one of the 33 positions in each library. Using the previously trained ML algorithm to predict the viability of each of these theoretical libraries, the importance of each degenerate amino acid position was evaluated. This led to the identification of critical residues with low tolerance to mutations. Producing mini-libraries containing either three critical or three non-critical degenerate residues finally demonstrated a higher viability of the non-critical mini-library, thereby verifying the importance of the critical residues identified by the ML approach.

### 4.2. Ongoing Ventures in ML-Based Engineering of AAVs

Next to the two peer-reviewed studies on the use of ML for AAV vector engineering described above, several recent pre-print studies followed similar goals [189,190,191]. Of note, Sinai et al. [189] followed up on the study of Bryant et al. [187] using an unsupervised approach. Unsupervised ML algorithms do not depend on the “supervised” annotation of training datasets but rather work with non-annotated data. In this case, Sinai et al. employed evolutionary information of multiple-sequence alignments from AAV2-related viruses (per definition, these naturally occurring variants are considered as viable capsids), which was augmented by the sequence information of viable capsid variants derived from a deep mutational exploration within the 560–588 positions. Through Variational Auto-encoder and Independent Sites models, the authors were able to predict viable capsids with up to 28 mutations distance from WT AAV2.

In an attempt to avoid the necessity of generating additional experimental input data entirely, Mikos et al. [190] employed 3D structural data from AAV2 and related viruses from the Protein Data Bank. By focusing on the microenvironment characteristics of each residue as training data for a Random Forest model, the authors identified 74 variable residues. When interrogating the fitness of these 74 mutable positions from the Ogden dataset [37], the predicted residues were found to mostly tolerate mutation.

Zhu et al. [191], on the other hand, focused on the AAV5 capsid with a 7mer peptide insertion in VR VIII instead of a substitution/single-insertion mutagenesis. Starting from a baseline “NNK” 7mer library, the authors used weighted enrichment scores between plasmid and viral libraries as training data for linear models as well as neural networks. Focusing systematically on a trade-off between predicted enrichment (i.e., library viability) and diversity, the authors used model-guided library design demonstrating that at some point, a higher library viability will come at the cost of significant diversity loss. Zhu et al. experimentally validated their results by cloning an optimal trade-off library with high diversity and high viability using the model-predicted marginal probabilities for each nucleotide at each position (instead of using the NNK nucleotides across the 7mer stretch). The resulting library yielded five-fold higher titers than the NNK library and exhibited a much greater diversity after both, viral packaging and subsequent transduction followed by rescue from primary human brain tissue, exemplifying its superior fitness for downstream selections.

While the initial focus of ML-based approaches for AAV capsid and library design was mostly directed towards capsid viability, i.e., the ability to efficiently assemble and package its own genomes, this primary selection of a capsid variant within a library is only the first task in a long line of requirements for successful gene therapy vectors. Downstream tasks such as Nab evasion, low immunogenicity, efficient on-target transduction and reduced off-targeting are equally important [192,193]. With the generation of more viable libraries, the success of finding capsids that fulfill these tasks is greatly improved. ML approaches may assist in downstream selection tasks as well by enabling closed-loop engineering workflows. Such workflows would consist of repeated synthetic library generation, enrichment (e.g., capsid production, in vivo selection or Nab evasion), subsequent ML-based variant ranking and library design [192]. If successfully implemented, closed-loop engineering circuits may transform future capsid design endeavors and allow a more data-driven and deeper exploration of the AAV sequence space.

## 5. Concluding Remarks

Over the past decades, research in academia and industry has led to a rich repertoire of viral vectors, therapeutic antibodies, targeting ligands and oligonucleotide-based therapies that all found a therapeutic niche and were employed to tackle diseases. It is intriguing how especially AAV vectors have served as scaffolds for high-throughput capsid modification, chemical conjugation or genetic fusion of a plethora of molecules, from oligonucleotides or ligands to therapeutic proteins.

More recently, the field has witnessed a shift from the classical random to data-driven diversification approaches coupled with more sensitive and machine-driven interrogation. This was supported by the rich history of AAV capsid engineering with all its successes and failures that offers a unique data repertoire for ML approaches. In the future, ML may even enable in silico prediction of functional AAV candidates and thereby help to circumvent the limitations and pitfalls of classical *cap* gene engineering, such as species specificity and translatability. This, together with novel rational engineering efforts, such as the fusion or coupling of nanobodies or DARPins, might (re)shape the future of not only AAV, but any viral vector-based approach. It is important to note that both trends have profited from fundamental insights into AAV and host biology, especially the identification of potentially beneficial cellular receptors and matching targeting moieties, which underlines the importance and pivotal contribution of basic biology research.

One important topic that we could not elaborate on due to space reasons is the impact of bioanalytical methods as key determinants in the process of identifying promising AAV candidates. Technologies such as quantitative (q)PCR, NGS, single-cell sequencing and mass spectrometry methods allow one to follow the flow of genetic information within a biological system in a high-throughput manner and, if required, on a single-cell level from DNA, to RNA, to protein expression [23,26,27,28,76,194,195]. These screening methods are not only valuable for the endpoint analysis of pre-selected candidates but have also recently been combined with in vitro or in vivo selection [68,76,89], which significantly improved the identification of more promising and cell-type specific AAVs within complex viral libraries and biological systems.

Collectively, we believe that this upcoming next generation of parvoviral vectors will expand gene therapy applications beyond rare monogenic conditions in the future, to become standalone or combinatorial tools in the fight against common illnesses from infectious diseases to cancer as well as in immunotherapy.

## Data Availability

Not applicable.
